# Photonic reagents for concentration measurement of flu-orescent proteins with overlapping spectra

**DOI:** 10.1038/srep25827

**Published:** 2016-05-16

**Authors:** Alexei Goun, Denys I. Bondar, Ali O. Er, Zachary Quine, Herschel A. Rabitz

**Affiliations:** 1 Department of Chemistry, Princeton University, Princeton, NJ 08544, USA

## Abstract

By exploiting photonic reagents (i.e., coherent control by shaped laser pulses), we employ Optimal Dynamic Discrimination (ODD) as a novel means for quantitatively characterizing mixtures of fluorescent proteins with a large spectral overlap. To illustrate ODD, we simultaneously measured concentrations of *in vitro* mixtures of Enhanced Blue Fluorescent Protein (EBFP) and Enhanced Cyan Fluorescent Protein (ECFP). Building on this foundational study, the ultimate goal is to exploit the capabilities of ODD for parallel monitoring of genetic and protein circuits by suppressing the spectral cross-talk among multiple fluorescent reporters.

The development of fluorescent proteins (FPs) has revolutionized the life sciences. These proteins, used as fluorescent reporters of gene expression and biological function, have enabled qualitative as well as quantitative *in vivo* studies of genetic and protein network dynamics^1^. As a result, fluorescent proteins are extensively used in most modern biological laboratories. The standard practice for interrogating biological systems with FP reporters utilizes filtered excitation/emission spectroscopy to detect their expression. While this technique has proven extremely effective and produced a wealth of new results and applications, it is not without shortcomings. Most importantly, spectral crosstalk limits the number of FPs that can be simultaneously monitored in a single experiment, due to the broad overlap of emission and absorption spectra of different reporters^2^,^3^,^4^,^5^,^6^. Furthermore, while there are hundreds of types of FPs with unique characteristics suited to specific applications, the low photon yield of many of these reporters forces overexpression at concentrations that interfere with biological activity. Also, the absolute measurement of concentration is difficult, especially in biologically relevant conditions with low concentrations of FPs and high scattering through biological media, making these measurements primarily qualitative in nature. The ability to simultaneously, reliably, and quantitatively measure concentrations of several FPs could significantly advance a number of areas in the biological sciences, including synthetic biology^6^, neuroscience^7^, and cytometry^8^,^9^,^10^,^11^,^12^. In the current work, we take a first step towards this goal by employing *Optimal Dynamic Discrimination* (ODD)^13^,^14^,^15^,^16^,^17^,^18^,^19^,^20^: a scalable optical technique that operates on different physical principles from conventional linear excitation/emission spectroscopy. We demonstrate the capability of ODD to make reliable measurements of sub-micromolar concentrations of FPs with overlapping spectra. The experimental utility of ODD is illustrated by quantitatively characterizing mixtures of Enhanced Blue Fluorescent Protein (EBFP) and Enhanced Cyan Fluorescent Protein (ECFP) in cell extract.

ODD was originally inspired by theoretical analysis showing that nearly identical molecules may be distinguished by means of their quantum dynamics induced by properly shaped laser pulses^13^. This theory has been confirmed experimentally for a number of systems^14^,^15^,^16^,^17^,^18^,^19^,^20^. ODD differentiates near-identical molecules by actively driving the unique excited state dynamics of each species of molecules within the mixture using the coherent nonlinear interaction between the laser field and the molecule. This operation is in contrast to conventional linear fluorescence spectroscopy, which passively excites all molecules in a mixture and monitors the fluorescence emitted by the molecules as they randomly decay back to their ground states. In ODD, femtosecond laser pulses with tailored temporal structure interact with the molecules on the time scale of their natural motion: by controlling the shape of the laser pulse we may uniquely guide the excited state dynamics of the molecules in the sample. These interactions can be complex, even for the simplest molecules, and the laser pulse shapes that uniquely excite the molecules generally can not be determined from first principle calcuations. For this reason, we use closed-loop, adaptive feedback optimization in the laboratory to discover the specially shaped laser pulses that produce distinct responses from each FP within the mixture^13^,^16^,^20^,^21^,^22^. These optimally shaped pulses, or *Photonic Reagents*, allow us to draw apart the dynamic spectral signatures of species that would be very difficult to distinguish by means of linear spectroscopy^15^,^23^,^24^. Moreover, we may choose the feedback to the optimization such that the procedure not only increases the intensity of signals collected from the molecule of interest, but also enhances the accuracy of the concentration determination, thereby yielding more reliable characterization of sample composition. With adequate optical resources (i.e., broad bandwidth, stable, coherent radiation) the ODD technique may be extended to a much larger number of optical reporters than accessible by current techniques. These characteristics hold out the prospect of making ODD a valuable technique for enhancing biological characterization.

## The ODD Algorithm for concentration determination

Optimal Dynamic Discrimination is implemented in two stages. In the learning stage, a stochastic algorithm discovers an optimal series of photonic reagents where each yields distinguishable signals from pure samples of individual FPs. In the next stage, the discovered set of optimal photonic reagents can be applied to any mixture of the FPs to measure the component concentrations. The algorithm is described below for two FPs, and presented more generally for an arbitrary number of species in the Supporting Information.

### The Learning Stage: Photonic reagent optimization

The total fluorescence from a mixture of FPs is simply the sum of the fluorscence signals of each component, weighted by their respective relative concentrations. For a mixture of two FPs we have:


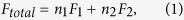


where *F*_1_ and *F*_2_ are the integrated fluorescence signals from reference samples of known concentration, and *n*_1_ and *n*_2_ are the unknown concentrations (relative to the reference samples) in the mixture. To determine these unknown concentrations the samples are excited by (at least) two different *photonic reagents* (*PR*_1_ and *PR*_2_), and the measured signals are arranged into the matrix below:


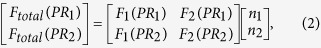


where the fluorescence intensities are a function of the photonic reagent used to excite the samples. The values of the known reference concentrations normalize the measured fluorescence signals; in this way they are incorporated in the final determination of the unknown concentrations.

The system of linear equations in Eq. (2) has a unique solution when the determinant of the matrix of measured reference fluorescence intensities (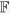) is non-zero:


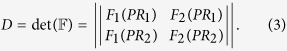


Furthermore, based on the Cramér-Rao inequality^25^, the error in the concentration determination is inversely proportional to the magnitude of |*D*|. Therefore, we perform stochastic optimization to find an optimal pair of photonic reagents which maximize |*D*|. Such an objective function has a degree of robustness to additive noise (e.g. shifting all fluorescence signals by a constant leaves *D* unchanged). Moreover, 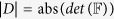 is a convex function of the matrix argument 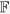, suggesting that the optimization procedure should be robust^22^. In the engineering literature such problems are related to what is called D-optimal experimental design^25^. Even though the unknown concentrations enter linearly in Eq. (2), the optimization problem is a non-linear function of the control field because the fluorescence depends non-linearly on the interrogating photonic reagents. The details of the experimental implementation are given below.

In order to experimentally discover an optimal pair of photonic reagents, we have developed the following revision of the conventional closed-loop adaptive algorithm^21^. We begin by generating 2*N* random photonic reagent pulses grouped into *N* pairs (i.e., iterations:)


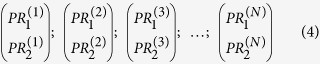


The upper index labels the photonic reagent iteration. In the current experiment, we employed *N* = 30. For each photonic reagent, 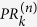, we record the fluorescence from the reference samples of known concentration: 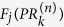 for *k*, *j* = 1, 2, and *n* = 1, …, *N*. This yields fluorescence intensities from 2*N* pulse shapes, while the objective function in Eq. (3) depends on only 2 pulses. Thus, we form all possible pairwise combinations from the recorded 2*N* measurements (1770 combinations) and then calculate and sort the objective functions, |*D*|, for these combinations to be used as feedback to the genetic algorithm (GA). This bundling of the individual photonic reagents into pairs and the subsequent unbundling and resorting of the measured fluorescences is a consequence of the nature of the GA used to discover the optimal combinations of photonic reagents. This procedure becomes more significant at higher dimensions, and is discussed in the Supporting Information. Based on the fitness of the previous generation, the GA determines new pulse shapes for each subsequent iteration, where the “genes” are the pulse shaper settings that modulate the complex temporal field of the laser pulse^21^. This procedure is iterated to discover the combination of photonic reagents that maximizes the objective function |*D*|.

In principle, this algorithm may be repeated until full convergence is reached; however, in practice the FPs photo-degrade if exposed to laser radiation for extended periods of time, even when circulated, yielding an increasingly uninformative signal at long optimization times. In the present experiments, we found that the algorithm returns reliable results even after a few iterations before significant photo-degradation occured. The degree to which photo-damage does occur was measured and corrected by comparing the reference solution fluorescence intensities at the beginning of the experiment to the intensities during and after optimization under equivalent excitation conditions. All results provided below are corrected for photo-degradation.

### The Application Stage: Measuring concentration with optimized photonic reagents

At the end of the learning stage, we have a final *N* pairs of photonic reagents ranked in descending order by the value of the objective function |*D*|, characterizing the accuracy of the concentration measurement. At this point, the top ranking pair of photonic reagents is the optimum solution, which is nominally sufficient to determine the sample concentrations within a mixture of FPs for analysis. However, to further increase the accuracy of the measured concentrations, we pick the *P* highest performing pairs (in our experiment, *P* = 10) and use them to interrogate the mixture, thereby collecting 2*P* measurements of the left hand side on the following system of equations:


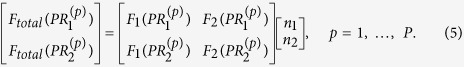


This is an overdetermined system of 2*P* measurements with just 2 unknowns, which we solved using the unconstrained least squares method with no assumption on values of *n*_*j*_. In cases where faster recording or lower light exposure is preferred, concentrations could be determined using as few as a single optimized pair of photonic reagents. The number of photonic reagent pairs used to characterize the mixture would depend on the desired accuracy of the concentrations and the experimental noise conditions of the measurements. This rather straightforward approach worked well in our experiments; however, if the absolute concentrations of FPs are very low, thereby decreasing the signal-to-noise ratio, other more advanced techniques may be needed to compensate for measurement noise. For example, constrained least squares fitting, D-MORPH regression^26^, Bayesian inference, etc. could be used to incorporate knowledge about the system, such as forbidding negative concentrations: *n*_*j*_ ≥ 0.

## Experimental Demonstration of ODD with fluorescent proteins

**Figure 1:**
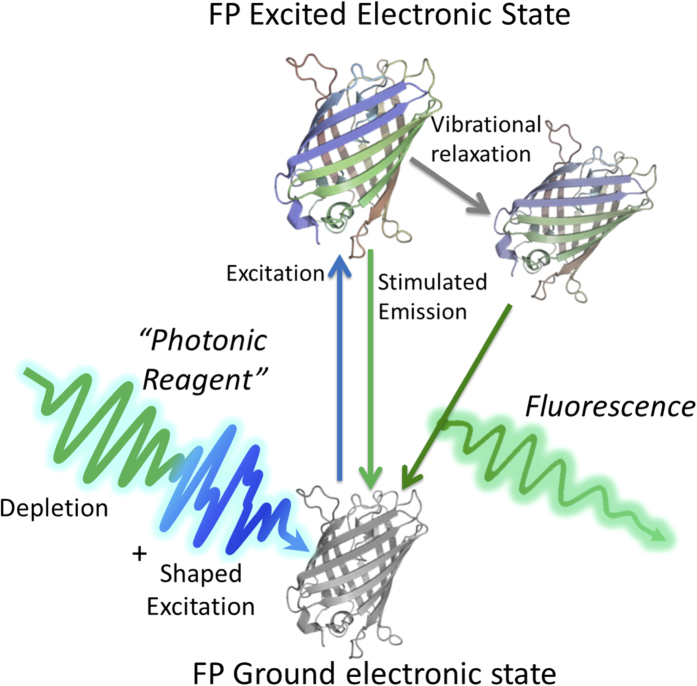
Physical mechanism of ODD: One *photonic reagent* is a combination of a shaped excitation pulse, which creates a unique coherent vibronic wave packet in each species of FP, and an unshaped stimulated depletion pulse. The dual sub-pulse sequence alters the fluorescence emission of the FP molecules, enabling discrimination of the particular FPs within the mixture.

**Figure 2:**
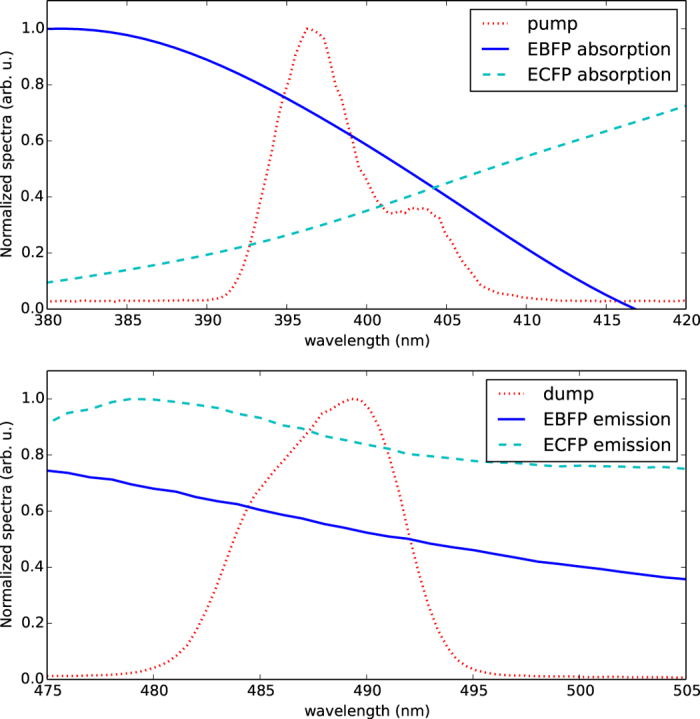
Absorption and emission spectra of EBFP and ECFP and the excitation pulse spectrum as well as the depletion pulse spectrum used in the ODD experiment.

The nonlinear optical interaction exploited by this implementation of ODD is represented by the schematic in Fig. 1. An FP molecule is interrogated by one photonic reagent, made up of a dual sub-pulse “*excitation-depletion*” sequence. The excitation portion of the photonic reagent is an optimally shaped ultrashort laser pulse, which coherently transfers the ground state population of each FP into a particular superposition of vibrational states in the first excited electronic level. This creates a coherent wave packet with tailored dynamics that influence its interaction with the later portion of the photonic reagent. This second portion is a longer wavelength, unshaped ‘depletion’ pulse, which is tuned to overlap the emission spectra of the FPs (see Fig. 2). The interaction of the excited wave packet with the depletion portion of the photonic reagent dictates whether a particular FP will be preferentially driven back down to the ground state *coherently*, or will remain in the excited state to decay at a later time by fluorescing. In this way, the final fluorescence signal of the FPs is altered after interacting with photonic reagents of equivalent spectral intensity but distinct tailored temporal structure. By collecting the response of a set of reference FP samples to several pairs of photonic reagents in the learning stage the shaped excitation pulse may be algorithmically optimized to maximally distinguish the individual components in the mixture^19^,^24^.

From an examination of the absorption spectra of EBFP and ECFP in Fig. 2, it is evident that these two specific FPs could be effectively distinguished using standard UV excitation/emission measurements; however their significant spectral overlap still makes them good candidates to test and develop the novel, scalable nonlinear optical capability of ODD. The ultimate utility of ODD lies in the multiplexing of several photonic reagents to expand the application of the technique to several FPs, beyond the limitations of conventional linear excitation/emission measurements. This multiplexed scaling rests on operating in the ultrafast coherent regime, where we can exploit the rich dynamics of the excited molecular states by drawing on the ability to create an arbitrary number of distinctly shaped interrogating photonic reagents. Interestingly, ODD will likely best operate in complementary applications to linear spectroscopy.

A similar nonlinear, excitation-depletion interaction is exploited in the super-resolution imaging technique known as Stimulated Emission Depletion (STED) microscopy^27^,^28^. However, the utilization of coherent stimulated emission in ODD is distinct from its application in STED microscopy. In STED, complete suppression of fluorescence is the goal, to maximize the contrast between the un-depleted sub-diffraction-limit spot and the depleted surrounding region. To accomplish this, the depletion pulse arrives *after* vibrational decoherence has transfered all excited molecules into the lowest vibrational state of the excited electronic level, and it is typically tens to hundreds of picoseconds long, allowing it to interact with the molecule until the excited state is completely depleted. By contrast, ODD depletes the excited state *before* decoherence of the vibrational wave packet, forcing the depletion pulse to be very short and nearly simultaneous with the excitation pulse. This reduces the maximum achievable depletion, but grants access to the highly species-specific information carried in the rich motion of the coherent excited vibronic wave packet. Because ODD only requires that each FP in a mixture respond in a unique way to a particular photonic reagent, total excited state depletion is not necessary for successful descrimination.

**Figure 3:**
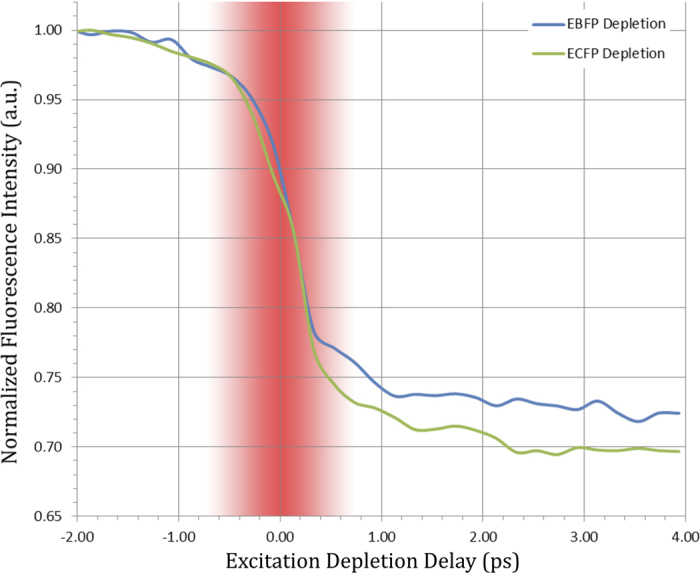
Depletion curves (using unshaped excitation) for EBFP and ECFP in cell extract. The shaded region around *t* = 0 indicates the domain where the excitation and depletion pulses overlap, allowing effective manipulation of fluorescence by shaping of the complex phase of the excitation pulse.

Because of the extremely short lifetime of the excited vibrational wave packet (typically on the order of ~1 ps) the relative timing between the excitation and depletion pulses is a critically sensitive parameter in ODD. The dependence of the fluorescence on this time delay with no shaping of the excitation pulse is shown in Fig. 3 for EBFP and ECFP in cell extract. Both curves are normalized to the total fluorescent emission intensity without depletion, and *t* = 0 on the figure corresponds to synchronization of the centers of the excitation and depletion pulses. As shown in the figure, the depletion curve can be split into three time domains. When the depletion pulse follows the excitation pulse by longer than the coherence lifetime of the vibrational wavepacket (positive delays ⪆0.5 ps) a steady state portion of the excited state population is stimulated down to the ground state. This circumstance will not exploit the species-specific sensitive information in the short-time dynamics created by the shaped excitation pulse. At much longer delays than shown in Fig. 3 (~nanoseconds) the molecules naturally decay to the ground state and the fluorescence returns to its undepleted intensity. At negative delays (beyond ~−0.5 ps in Fig. 3) the sample fluoresces as if solely exposed to the excitation, because the depletion pulse is outside the absorption spectrum of the ground state of both FPs. It is only at very short delay times, in the highlighted region between approximately −0.5 and 0.5 ps in Fig. 3, that there exists the unique dynamical window for effective coherent manipulation of fluorescence, as in that domain the photo-dynamics become sensitive to the phase structure of the laser pulses. This region is where the ODD algorithm can effectively exploit those dynamics. There is flexibility in setting the precise depletion delay, with each set of FPs exhibiting its own characteristics. For this experiment the initial delay was set to zero (excitation and depletion pulses synchronized) allowing the algorithmically guided pulse shaper to seek out the best delay as part of the optimization procedure.

### Experimental Apparatus

**Figure 4:**
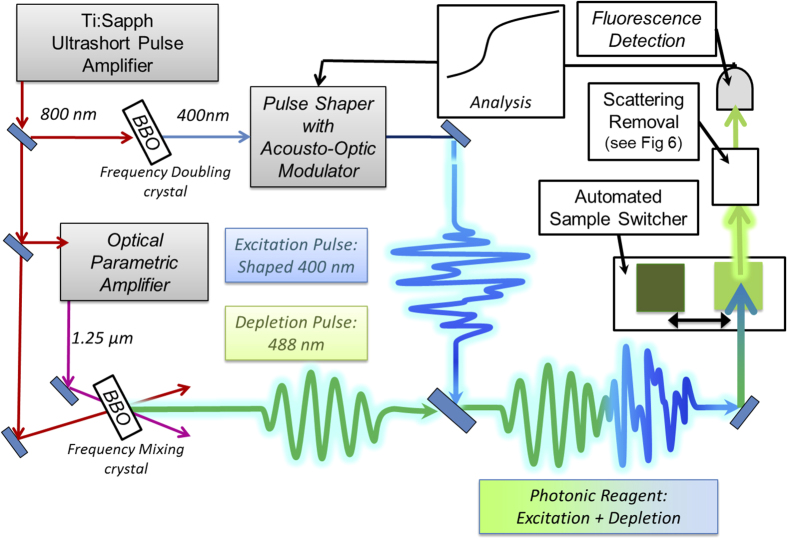
Schematic of experimental apparatus for ODD. The scattering removal system is further described in Fig. 5.

The present demonstration of ODD is based on a KHz system and not on a MHz laser typically employed for microscopy. The schematic of the experimental apparatus is shown in Fig. 4. The primary 800 nm laser pulses were generated by a Titanium:Sapphire ultrafast regenerative amplifier system (Coherent Legend) with a repetition rate of 1 kHz, average power of 2 W, and pulse duration ~40 fs. The source beam was split in two parts to make the excitation and depletion beams. The excitation beam was created by doubling the primary beam to 400 nm in a BBO crystal and the complex temporal structure of the pulse was constructed in a 4-F configuration pulse shaper with a computer controlled acousto-optic modulator^29^, which independently adjusts the phases and amplitudes of each spectral component of the pump beam. To generate the depletion beam, a portion of the primary amplifier output was used to drive a Quantronix variable wavelength Optical Parametric Amplifier (OPA) with an output at 1.25 *μ*m. This beam was mixed with the remainder of the primary 800 nm beam by sum frequency generation in a BBO crystal to obtain the desired 488 nm depletion pulse. A band pass filter (FL488-10, Thorlabs) was used to spectrally isolate this depletion beam from its parents.

The characteristics of the excitation and depletion pulses are given in Table 1. The pulse energies were chosen to maximize the stimulated depletion signal while avoiding undesirable power effects, such as self-focusing, extensive sample bleaching, or absorption from the excited state. The two beams are synchronized with a computer controlled delay stage and recombined on a dichroic mirror before passing through routing and focusing optics into the sample cuvettes. The excitation and depletion beams must overlap both spatially, at their shared focal point, and temporally, within the dynamic coherence window, to produce ODD coherent fluorescence depletion, necessary for achieving reliable concentration measurements. The beam waist of the depletion beam is spatially wider than the excitation beam, to ensure the entire population of excited FPs are exposed to the depletion pulse.

A special design feature of our experiment is an automated sample switcher configuration. Four quartz flow cells were fixed onto a computer controlled translation stage. The total integrated fluorescence from each sample was measured on a single photomultiplier tube while each sample was moved into the beam path. Two cells contain reference samples of the FPs with known concentration measured during the learning stage to discover optimal photonic reagents. The other two cells contain different mixtures, whose concentrations are to be characterized. All cells are connected to piezo pumps for circulation to minimize photo-bleaching. This sample setup has many advantages over a configuration where the laser beam is divided among multiple stationary cells. The setup utilized here not only exploits the full beam energy in each sample, but also ensures that the pulse is identical and not distorted by beam dividing optics. Additionally, this configuration occupies less space and is readily scalable to a larger number of sample cells. In order to obtain reliable concentration measurements, it is necessary that all samples are maintained in identical condition during laser field interrogation: the cells must be made of the same material and carefully aligned with respect to each other.

**Figure 5:**
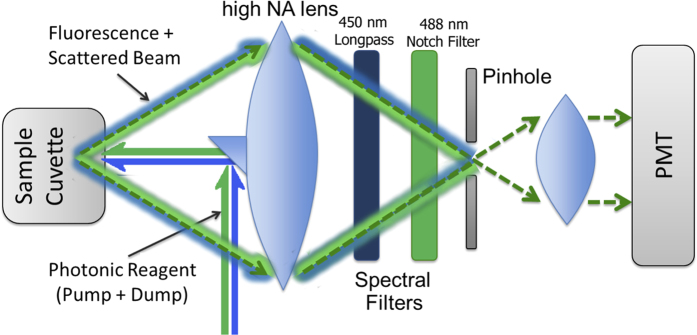
Fluorescence collecting system. A high NA lens collects the fluorescence signal from a large solid angle, while a small mirror attached to the lens reflects away the back reflected primary beam. A combination of spectral and spatial filtering (notch filter and pinhole) removes the remainder of the scattered primary beam. The sample is in close proximity to the lens front focal point such that the collected fluorescence is reasonably collimated enabling the operation of the notch filter.

Overcoming laser beam scattering in the cell extract was a major obstacle in measuring fluorescence intensity since the depletion beam spectrally overlaps with fluorescence. Figure 5 shows the setup to disentangle the (comparably) weak fluorescent signal from the bright background of scattered dump. Even though the depletion beam and fluorescence emission are spectrally alike, they have very different spatial characteristics. A high numerical aperture lens, is seen on the left of Fig. 5. At the center of this lens, a small mirror is glued to guide the control beam towards the sample and to block its reflection from the sample cell. The large numerical aperture lens collects the broadly emitted fluorescence along with the remaining scattered light. To improve the isolation of the fluorescence, a notch filter (NF488-15, Thorlabs) combined with a lowpass filter (FEL0450, Thorlabs) removes a substantial portion of the remaining scattered dump pulse without completely blocking the fluorescence. The final portion of the scattered laser is suppressed by spatial filtering with a pinhole, similar to the technique used in confocal microscopy, ensuring that the signal is collected only from the spatial point of the sample where the excitation and depletion beams meet. In this way, a clean depleted fluorescence signal is obtained.

We found that the signal-to-noise ratio of the fluorescence measurement in the *application stage* should be at least as high as during the *learning stage* of ODD to ensure accuracy. This was readily achieved by increasing the fluorescence acquisition time to 2 sec per measurement when interrogating the unknown mixtures, while 1 sec of acquisition was sufficient during the learning stage. Extending the acquisition time ensured that adequate signal would be collected from the unknown samples, whose component concentrations could be lower than the reference samples.

### Fluorescent Protein Samples

Lyophilized EBFP and ECFP were purchased from Biovision in 100 *μg* quantities and reconstituted in phosphate buffer. Quantum yields of EBFP and ECFP are 0.15 and 0.68, while the extinction coefficients are 1.9 · 10^5^ and 2.6 · 10^6^ (M^−1^cm^−1^), respectively. Each sample was diluted to 1 mL with 10 mM phosphate buffer, 140 mM NaCl, and 2.7 mM KCl at pH 7.4. The absorption and emission spectra of ECFP and EBFP are shown in Fig. 2.

Proteins absorbing in blue region were selected not only because they were convenient for our laser sources, but also because this regime is known to be challenging for biological imaging due to low quantum yields of the FPs. The success of the experimental implementation of ODD in this regime strongly suggests that such an approach will function well for other FPs operating at longer wavelengths.

The protein samples were studied in cell extract, to simulate a biologically relevant sample environment, and were prepared according to the following protocol: *E. Coli* BL21 DE3 cells were incubated with PCA24N plasmid, containing a control gene that expresses a small four-helix bundle protein, and transformed by electroporation. The transformed cells were plated on Luria-Bertani (LB) agar plates supplemented with 30 *μ*g/ml chloramphenicol (CAM) and grown overnight at 37 °C. A single fresh colony was used to inoculate a 1L flask of LB + CAM. The culture was grown to OD600 = 0.6 and induced with IPTG at a final concentration of 100 *μ*M. The culture was grown over night, and then centrifuged and frozen. Cell lysates/extracts were prepared by resuspending frozen cell pellets in 50 mls of 1X phosphate buffered saline at pH 7.4. Cells were then lysed on ice using an ultrasonic homogenizer set to 40% power with 5 seconds of sonication followed by 5 seconds of dwell time for a total of 10 minutes. The whole cell lysate was centrifuged at 8000 g’s for 30 mins. The supernatant cell extract was decanted and frozen in small aliquots for subsequent use in the experiments.

### Results of concentration measurements

Utilizing ODD, we analyzed a series of two-component mixtures of EBFP and ECFP dissolved in cell extract at biologically relevant concentrations. The results of these measurements are collected in Table 2.

**Figure 6:**
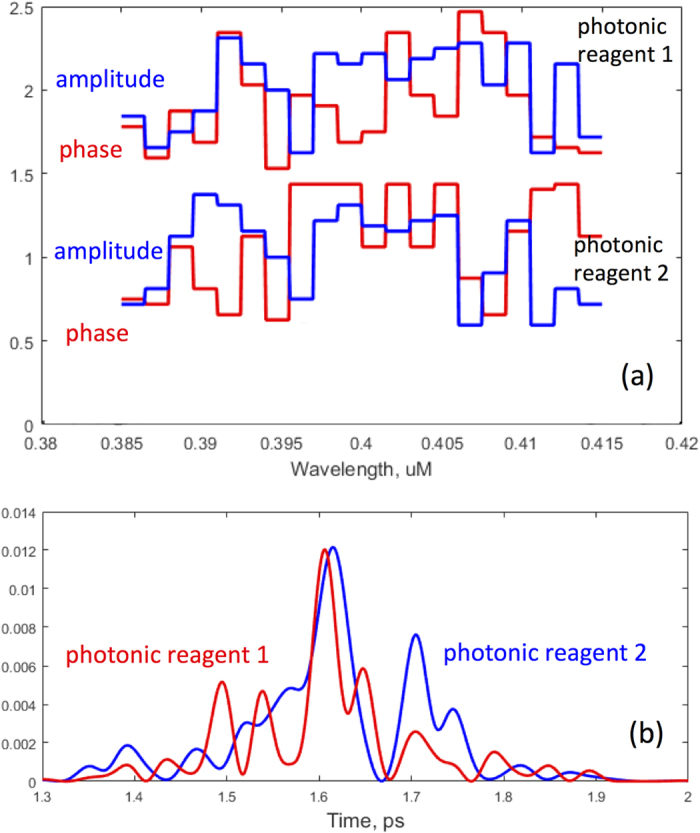
An example of shaped excitation pulses pairs used to characterize a mixture of EBFP and ECFP. (**a**) The normalized amplitude and phase masks for the photonic reagents. The solid line is the excitation pulse spectrum with the flat amplitude mask (see also Fig. 2). (**b**) The corresponding pulses in time domain.

A typical pair of photonic reagents *PR*_1_ and *PR*_2_ utilized to characterize the protein mixture [see equations (2) and (3)] are shown in Fig. 6. The instantaneous pulse intensity [Fig. 6(b)] are obtained as the Fourier transform of the recorded masks [Fig. 6(a)]. Extracting the physical mechanism for discrimination is a subject of our current investigation.

The errors in Table 2 were calculated as the standard deviation of five consecutive concentration measurements. Knowing the absolute concentrations of the reference solutions allowed us to translate the measured relative mixing ratios into absolute concentrations. The reference solutions were prepared by diluting the stock cell extract to micromolar concentration and characterized by UV excitation/emission spectroscopy. The EBFP and ECFP reference solutions were, respectively, prepared at a concentration of 3.20 *μ*M and 3.40 *μ*M. Four mixed samples were prepared from these reference solutions and characterised by ODD. In all cases, ODD reliably measured the absolute concentrations of the fluorescent proteins in the mixtures to high accuracy in spite of their overlapping spectra.

## Future Prospects for ODD

Using EBFP and ECFP as examples, we have demonstrated that ODD successfully discriminates and accurately measures concentrations for mixtures of FPs with significantly overlapping absorption/emission spectra. This operation was accomplished in a solution of *E. Coli* cell extract, establishing the suitability of ODD for interrogation of environments typically encountered in biological samples. The true utility of ODD does not lie in discriminating two FPs (e.g., as here, where linear spectroscopy could function), but rather in its scalability to draw on unique coherent FP excitation by utilizing a nearly endless set of distinct photonic reagents. To further advance ODD, we are currently reducing the complexity of the experimental system by replacing the ‘excitation-depletion’, multi-laser system with a single Non-colinear Optical Parametric Amplifier (NOPA), which is an ultra-broadband coherent light source with a bandwidth covering the entire visible spectrum. This flexible radiation source can create both the excitation and depletion pulses wrapped together, with the full absorption and emission bands of several FPs within its spectral range. Future studies should also be able to extend the capabilities of ODD by fully shaping the photonic reagents, building optical structure into both the excitation and depletion pulses, thereby allowing the photonic reagents to exploit more complex FP dynamics. These capabilities should enable more accurate, reliable, simultaneous detection of a larger number of FPs while greatly simplifying the technique.

## Additional Information

**How to cite this article**: Goun, A. *et al.* Photonic reagents for concentration measurement of fluorescent proteins with overlapping spectra. *Sci. Rep.* **6**, 25827; doi: 10.1038/srep25827 (2016).

## Supplementary Material

Supplementary Information

## Figures and Tables

**Table 1:** Laser pulse parameters.

	Excitation	Depletion
Center wavelength (nm)	400	488
Typical pulse width (ps)	~0.03–1.0	0.1
Peak intensity (GW/cm^2^)	4.3–142	19.8
Beam diameter (*μ*m)	100	170
Pulse energy (*μ*J)	0.3	0.4

The typical excitation pulse width depends on the phase-amplitude mask of the pulse shaper, and ranges from a transform limited width of 30 fs to the maximum shaper range of ~1 ps.

**Table 2:** Results of ODD. The first column contains the prepared mixing ratios of the FPs.

Mixtures of	Mixing Ratio	Concentration (*μ*M)
EBFP/ECFP	Measured by ODD	Measured by ODD
50–50%	(47 ± 2)−(53 ± 2)%	1.50 ± 0.06/1.80 ± 0.07
75–25%	(74 ± 1)−(26 ± 1)%	2.37 ± 0.03/0.88 ± 0.03
0–100%	(4 ± 8)−(96 ± 6)%	0.1 ± 0.3/3.3 ± 0.2
100–0%	(96 ± 1)−(4 ± 1)%	3.07 ± 0.03/0.14 ± 0.03

The second and third columns contain results of concentration determination with ODD, for the mixing ratio and absolute concentrations, respectively.
