# Supplementary material for: Photonic reagents for concentration measurement of flu-orescent proteins with overlapping spectra
Source: Sci Rep. 2016 May 16;6:25827. doi: 10.1038/srep25827 (PMC4867436; doi:10.1038/srep25827)
Supplement: Supplementary Information [file srep25827-s1.pdf]

Supplementary Information for "Photonic reagents for concentration measurement of fluorescent proteins with overlapping spectra": General ODD Algorithm for detecting an arbitrary number of species

Alexei Goun[1], Denys I. Bondar[1], Ali O. Er[1], Zachary Quine[1], Herschel A. Rabitz[1]

[1]*Department of Chemistry, Princeton University, Princeton, NJ 08544, USA*

The following material is a generalized description of the algorithm presented in Sec. II of the main text. The ODD algorithm is scalable, drawing on an essentially endless number of distinct interrogating photonic reagents. This enables the discrimination and quantification of the individual components of a mixture by variations of their optical response to a series of photonic reagents. This optical response is not limited to the fluorescence depletion measurement used in this work, and only requires that the measured observable respond uniquely to the photonic reagent control parameters: the spectral phase and amplitude of the interrogating laser pulse. The complex pulse shapes of the tailored photonic reagents are obtained by iterative optimization of the solution to the inversion problem of concentration determination. The objective function of this optimization is defined as follows.

The total optical response from an M-component mixture is a linear combination of the independent optical responses F of each species j to a *photonic reagent* PR_k, weighted by the

relative concentrations n_j of the species in the mixture:

$$F_{total}(PR_k) = \sum_{j=1}^{M} n_j F_j(PR_k), \quad k = 1, \ldots, M. \tag{1}$$

Because the signals of the individual species are independant they add linearly, but the measured optical responses are nonlinear functions of the control variables of the photonic reagent pulse.

The identities in equation (1) form a system of M linear equations for M unknown concentrations n_j. Such a system has a unique solution when the responses to the sequence of photonic reagents are distinct, making the following determinant non-zero:

$$D = \det(\mathbb{F}) = \left\| \begin{matrix} F_1(PR_1) & F_2(PR_1) & \cdots & F_M(PR_1) \\ F_1(PR_2) & F_2(PR_2) & \cdots & F_M(PR_2) \\ \vdots & \vdots & & \vdots \\ F_1(PR_M) & F_2(PR_M) & \cdots & F_M(PR_M) \end{matrix} \right\| . \tag{2}$$

where $\mathbb{F}$ is a matrix of measured fluorescence intensities: each element $\mathbb{F}_{jk}$ is the optical response from a reference sample of species j with known concentration C_j exposed to PR_k.

Based on the Cramér-Rao inequality (see, e.g., Ref. [25]), the error in the concentration determination is inversely proportional to the magnitude of this determinant: $|D|$. The larger the magnitude of D, the higher we expect the accuracy of the determined concentrations; therefore, this measurement is an effective objective function, or fitness score, to an iterative stochastic optimization algorithm to maximize $\mathrm{abs}\,(D)$. Such an objective function has a degree of robustness to additive noise (e.g. shifting all fluorescence signals F_j by a constant leaves D unchanged). Moreover, $\mathrm{abs}\,(\det(\mathbb{F}))$ is a convex function of the matrix argument $\mathbb{F}$, suggesting that the optimization

procedure should be robust [22]. In the engineering literature such problems are related to what is called D-optimal experimental design [25].

In order to discover an optimal M-tuple of photonic reagents in our experiments, we developed a custom closed-loop adaptive algorithm [21]. We begin by generating N random samples of M-tuples of photonic reagents ($PR_m^{(n)}$):

$$\begin{aligned}
&\left(PR_1^{(1)},\, PR_2^{(1)},\, \ldots,\, PR_M^{(1)}\right);\\
&\left(PR_1^{(2)},\, PR_2^{(2)},\, \ldots,\, PR_M^{(2)}\right);\\
&\vdots\\
&\left(PR_1^{(N)},\, PR_2^{(N)},\, \ldots,\, PR_M^{(N)}\right). \qquad (3)
\end{aligned}$$

The upper index labels the photonic reagent iteration. In the current experiment, we employed $N = 30$ and $M = 2$; however the ODD procedure is designed to be scalable and we are extending the method to a larger number of fluorescent proteins ($M \sim 10$).

For each photonic reagent, $PR_k^{(n)}$, we record the fluorescence from the reference samples: $F_j\left(PR_k^{(n)}\right)$ for $k, j = 1, \ldots, M$, and $n = 1, \ldots, N$. Since the information about the fluorescence from NM pulse shapes is available while the objective function in Eq.(2) depends only on M pulses, we form all possible combinations of M out of the recorded NM pulses, calculate the objective functions, and sort the results by magnitude:

$$|D\left(PR_{k_1}^{(n_1)}, PR_{k_2}^{(n_2)}, \ldots, PR_{k_M}^{(n_N)}\right)|, \qquad (4)$$

where each k_i takes on the value $k_i = 1, \ldots, M$ and each n_j takes on the value $n_j = 1, \ldots, N$.

The multiplicity of this set of calculations is

$$\binom{NM}{M} = (NM)!/[M!(NM-M)!]. \tag{5}$$

Out of this sorted set of pulse sequences, we pick new M-tuples based on the largest values of the objective function.

The optimization algorithm is a stochastic genetic algorithm modeled on biological evolution, where a single genome string made up of the pulse shaper settings [21] is assigned a fitness score, the objective function in Eq.(2). This fitness score dictates how the controls advance and combine in the subsequent iterations to improve the response of the photonic reagents, maximize $\mathrm{abs}\,(D)$, and improve the concentration determination.

The M pulse shapes of each photonic reagent in the M-tuple of a given iteration is concatenated into a single string to be processed by the GA, then re-separated and sent to the pulse shaper to tailor the laser pulses that interrogate the sample. However, it is possible that combinations of photonic reagents from different M-tuples would yield higher fitness interactions than those within a group, for example:

$$\left| D\left(PR_1^{(D)}, PR_2^{(A)}, PR_3^{(B)}, \ldots, PR_M^{(A)}\right) \right| > \left| D\left(PR_1^{(A)}, PR_2^{(A)}, PR_3^{(A)}, \ldots, PR_M^{(A)}\right) \right|. \tag{6}$$

This cross breeding across different iterations within a generation allows for higher fidelity solutions and even faster optimization in fewer generations at the expense of increased computational cost per generation.

After a number of iterations the optimization may be halted when an acceptable value of

the objective function is reached. The final generation of the optimization produces N samples of M-tuples of photonic reagents ranked in descending order by the value of the objective function, characterizing the accuracy of the concentration measurement of each M-tuple group.

At this point, the top ranking M-tuple of photonic reagents is the optimum solution and nominally sufficient to determine the sample concentrations of any combination of the characterized species. However, to further increase accuracy of the measured concentrations, one may pick the P highest performing M-tuples and use them to interrogate the mixture. Beyond the statistical enhancement of repeated sample interrogation, each photonic reagent M-tuple has evolved to manipulate a different set of coherent dynamics in the species. We collect P measurements of the left hand side of the following system of equations:

$$F_{total}\left(PR_k^{(n)}\right) = \sum_{j=1}^{M} n_j F_j \left(PR_k^{(n)}\right), \qquad k = 1, \ldots, M, \qquad n = 1, \ldots, P. \tag{7}$$

This yields an overdetermined system of PM linear equations to be solved for the M unknown n_j n_j's. The number of M-tuples (P) used to characterize the mixture will be determined by experimental signal to noise ratios, number of species being characterized, and desired concentration accuracy.
